# Evaluation of treatments for HIV-associated Kaposi sarcoma in Africa

**DOI:** 10.1186/s13027-021-00364-5

**Published:** 2021-04-17

**Authors:** Susan E. Krown, Margaret Z. Borok

**Affiliations:** 1 Member Emerita, Memorial Sloan-Kettering Cancer Center, New York, NY USA; 2 Department of Medicine, Parirenyatwa Hospital, College of Health Sciences, University of Zimbabwe, Mazowe Street, Harare, Zimbabwe

**Keywords:** Kaposi sarcoma, Human immunodeficiency virus, Chemotherapy, Paclitaxel, Pegylated liposomal doxorubicin

To the editors:

We write to comment on the recent article by Coldiron and colleagues [1] that describes Médecins Sans Frontières’ programmatic use of pegylated liposomal doxorubicin (PLD) for treatment of HIV-associated Kaposi sarcoma (KS) in Mozambique. Their discussion raises the important and very pertinent issue of health systems barriers to effective and sustainable cancer treatment in low- and middle-income countries, which is a problem for all cancers, not just for KS. That said, we wish to register our concerns about some of the particulars of their study and their interpretation of the data.

1. Response to treatment: The authors cite an overall tumor response rate of 80% and a complete response (CR) rate of 13%. These response rates, and the CR rate in particular, are higher than would be expected even in highly resourced settings where the adverse socioeconomic factors the authors cite as reasons for their high mortality rate are not present. This raises concerns about the rigor with which KS response and progression-free survival were documented. Response of KS was measured infrequently (every 3 months in the first year, and then every 6 months), and response assessment was described as consisting of measurement of “sentinel KS lesions”, which is only one of several measures required for a full KS response assessment. In addition, the duration of response was not specified.

Of study participants who achieved a response to PLD and whose chemotherapy was then discontinued, 28% required re-treatment after subsequent KS progression. In our experience, recurrence or progression of KS after apparently successful chemotherapy is not uncommon, nor is response to a subsequent course of treatment. We are, however, puzzled by the authors’ statement that “this phenomenon does not seem to be related directly to PLD”, as most agree that chemotherapy is not expected to cure KS, and that the cause of progressive KS is likely multifactorial. Nonetheless, it is disappointing that the investigators did not track ART adherence and virological failure, which are likely contributing factors to KS progression.

2. Safety: Except for blood counts performed prior to each chemotherapy cycle, formal assessments of safety and adverse events were done infrequently during the course of treatment. Other than blood counts, it is not stated whether any other tests to monitor safety (e.g., renal or hepatic function, cardiac function) were conducted routinely, and participants were apparently questioned about adverse events only at the times of their 3- to 6-monthly KS evaluations. Thus, it is not surprising that the reported rates of moderate to severe toxicities were lower than those reported from studies of PLD conducted in highly-resourced settings [2, 3]. Moreover, only significant adverse events (i.e., death or hospitalization) were described in detail, but details about other adverse events were not provided.

In this context, we highlight the authors’ comment regarding a prospectively randomized trial of chemotherapy with antiretroviral therapy (ART) in advanced KS [4], which we co-chaired. Our trial was conducted primarily in sub-Saharan Africa, and included frequent and intensive monitoring of KS response, safety, ART adherence and virologic control. Their comment that in our trial, “paclitaxel toxicities were largely the same compared to the vincristine/bleomycin group” seems to suggest that this was a failing of paclitaxel and that paclitaxel therefore suffers by comparison to PLD, whereas in fact the serious adverse event rate was low in both the paclitaxel and bleomycin/vincristine treatment arms of our trial.

We have additional concerns about participant screening. Formal assessment of cardiac function was done rarely (5 of 116 participants), and echocardiography was only performed if symptoms of CHF were detected. While we appreciate that the risk of cardiotoxicity is lower with PLD than with conventional doxorubicin, symptoms may be an unreliable indicator of cardiac dysfunction in this vulnerable patient population. For example, as noted in a recent review [5], although the incidence of HIV-associated cardiomyopathy has decreased from the pre-ART era, “the phenotype of cardiomyopathy has also changed markedly, from symptomatic systolic dysfunction … to asymptomatic systolic or diastolic dysfunction detected by echocardiography” in the post-ART era. Additionally, we are concerned about the infrequency with which the diagnosis of KS was pathologically confirmed (43%). As noted by Amerson et al [6], a clinical suspicion of KS is often not confirmed by pathology, and may be particularly challenging in people with darkly pigmented skin, so lack of a confirmed diagnosis risks unnecessarily exposing patients without KS to cytotoxic drugs.

3. Mortality: Overall mortality and, especially, loss to follow-up were high in this study. The loss to follow-up rate of 13% compares unfavorably with the < 1% we observed in our randomized trial [4], and at least some of those “lost” are likely to have died [7].

In summary, while we applaud these investigators for attempting to provide improved treatment for patients with advanced HIV-associated KS, we urge caution in interpreting the results presented. We suggest a cautious approach to patient screening prior to initiating chemotherapy, and careful attention to response assessment, safety monitoring and control of HIV and concomitant illnesses during therapy. Finally, we agree that a rigorously controlled, prospective, head-to-head comparison of PLD and paclitaxel in Africa is warranted. Such a trial is being planned, which will include not only clinical and safety endpoints, but also formal evaluations of quality of life as well as cost- and cost-effectiveness comparisons.

Susan E. Krown

London, United Kingdom

Margaret Z. Borok

Faculty of Medicine and Health Sciences, University of Zimbabwe, Harare, Zimbabwe

## Data Availability

Not applicable. No original data or materials presented.

## Abbreviations

ART: Antiretroviral therapy
CR: Complete response
HIV: Human immunodeficiency virus
KS: Kaposi sarcoma
PLD: Pegylated liposomal doxorubicin
